# *catena*-Poly[[diaqua­bis­(formato-κ*O*)cobalt(II)]-μ_2_-2,6-bis­(pyridin-4-yl)-4,4′-bipyridine-κ^2^ *N* ^2^:*N* ^6^]

**DOI:** 10.1107/S1600536811021118

**Published:** 2011-06-11

**Authors:** De-Yun Ma, De-En Sun, Guo-Qing Li

**Affiliations:** a School of Chemistry and Chemical Engineering, South China University of Technology, Guangzhou 510640, People’s Republic of China

## Abstract

In the title complex, [Co(CHO_2_)_2_(C_20_H_14_N_4_)(H_2_O)_2_]_*n*_, the Co^II^ ion, lying on an inversion center, is six-coordinated by two O atoms from two monodentate formate ligands, two N atoms from two 2,6-bis­(pyridin-4-yl)-4,4′-bipyridine (4-pybpy) ligands and two water mol­ecules, displaying an octa­hedral geometry. The 4-pybpy ligand, having a twofold rotation axis, functions in a bridging coordination mode, connecting the Co^II^ ions into a corrugated chain along [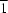
01]. The chains are further linked into a three-dimensional supra­molecular network by O—H⋯O, C—H⋯N and C—H⋯O hydrogen bonds and π–π stacking inter­actions between the pyridine rings [centroid-to-centroid distance = 3.743 (2) Å].

## Related literature

For general background to complexes with 2,6-bis­(4-pyrid­yl)-4,4′-bipyridine, see: Liu *et al.* (2009 ▶); Yoshida *et al.* (2007 ▶).
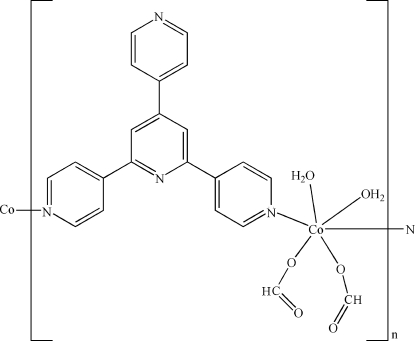


## Experimental

### 

#### Crystal data

[Co(CHO_2_)_2_(C_20_H_14_N_4_)(H_2_O)_2_]
*M* *_r_* = 495.35
Monoclinic, 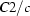

*a* = 24.412 (5) Å
*b* = 11.073 (2) Å
*c* = 7.4117 (15) Å
β = 91.28 (3)°
*V* = 2003.0 (7) Å^3^
*Z* = 4
Mo *K*α radiation
μ = 0.91 mm^−1^
*T* = 293 K
0.30 × 0.26 × 0.21 mm

#### Data collection

Rigaku/MSC Mercury CCD diffractometer
Absorption correction: multi-scan (*CrystalStructure*; Rigaku/MSC, 2002 ▶) *T*_min_ = 0.075, *T*_max_ = 0.126
9417 measured reflections
2303 independent reflections
1544 reflections with *I* > 2σ(*I*)
*R*_int_ = 0.063

#### Refinement

*R*[*F* ^2^ > 2σ(*F* ^2^)] = 0.044
*wR*(*F* ^2^) = 0.112
*S* = 1.11
2303 reflections
160 parameters
3 restraints
H atoms treated by a mixture of independent and constrained refinement
Δρ_max_ = 0.76 e Å^−3^
Δρ_min_ = −0.57 e Å^−3^

### 

Data collection: *CrystalStructure* (Rigaku/MSC, 2002 ▶); cell refinement: *CrystalStructure*; data reduction: *CrystalStructure*; program(s) used to solve structure: *SHELXS97* (Sheldrick, 2008 ▶); program(s) used to refine structure: *SHELXL97* (Sheldrick, 2008 ▶); molecular graphics: *SHELXTL* (Sheldrick, 2008 ▶) and *Mercury* (Macrae *et al.*, 2008 ▶); software used to prepare material for publication: *SHELXTL*.

## Supplementary Material

Crystal structure: contains datablock(s) I, global. DOI: 10.1107/S1600536811021118/hy2424sup1.cif

Structure factors: contains datablock(s) I. DOI: 10.1107/S1600536811021118/hy2424Isup2.hkl

Additional supplementary materials:  crystallographic information; 3D view; checkCIF report

## Figures and Tables

**Table 1:** Hydrogen-bond geometry (Å, °)

*D*—H⋯*A*	*D*—H	H⋯*A*	*D*⋯*A*	*D*—H⋯*A*
O1*W*—H1*W*⋯O2^i^	0.82 (1)	1.97 (1)	2.775 (3)	166 (4)
O1*W*—H2*W*⋯O2^ii^	0.82 (1)	1.99 (1)	2.814 (3)	174 (3)
C2—H2⋯N3^iii^	0.93	2.62	3.458 (3)	150
C5—H5⋯O2^ii^	0.93	2.53	3.429 (4)	164

Symmetry codes: (i) 
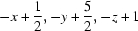
; (ii) 
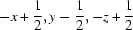
; (iii) 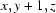.

**Fig. 1.**
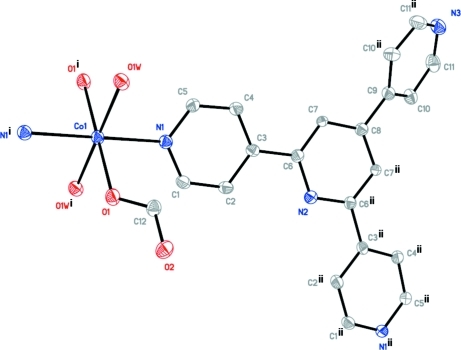
The structure of the title compound, showing 30% probability displacement ellipsoids. H atoms were omitted for clarity. [Symmetry codes: (i) 1/2 - x, 5/2 - y, -z; (ii) -x, y, 1/2 - z.]

**Fig. 2.**
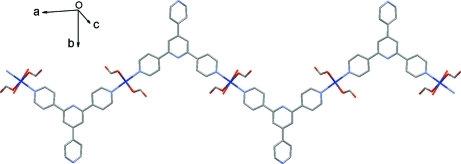
View of the corrugated chain. H atoms were omitted for clarity.

**Fig. 3.**
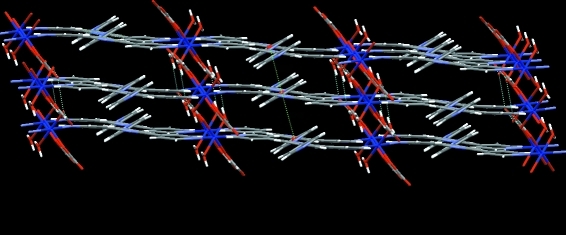
Crystal packing of the title compound. Hydrogen bonds and π–π stacking interactions are shown as dashed lines.

## supplementary crystallographic information

### Comment

In the structural investigation of 2,6-bis(4-pyridyl)-4,4'-bipyridine (4-pybpy) complexes, it has been found that 4-pybpy functions as a multidentate ligand (Liu *et al.*, 2009; Yoshida *et al.*, 2007), with versatile binding and coordination modes. In this paper, we report the crystal structure of the title compound, a new Co^II^ complex obtained by the reaction of 4-pybpy, cobaltous nitrate hexahydrate in DMF solution.

As depicted in Fig. 1, the Co^II^ atom, lying on an inversion center, is coordinated by two O atoms from two formate anions, two N atoms from two 4-pybpy ligands and two water molecules. The coordination environment of the Co^II^ atom can be described as octahedral. The 4-pybpy ligands link Co^II^ ions, forming infinite chains, with a Co···Co separation of 12.835 (2) Å (Fig. 2). These chains are further self-assembled into a three-dimensional supramolecular network *via* intermolecular O—H···O and C—H···O hydrogen bonds (Table 1) involving the water molecules as donors and O atoms of the formate groups as acceptors, and π–π stacking interactions between the pyridyl rings of neighboring 4-pybpy ligands (Fig. 3). The centroid–centroid distance is 3.743 (2) Å.

### Experimental

A mixture of 4-pybpy (0.155 g, 0.5 mmol), cobalt nitrate hexahydrate (0.145 g, 0.5 mmol) and DMF (15 ml) was placed in a 23 ml Teflon-lined reactor, which was heated at 358 K for 2 d, and then cooled to room temperature at a rate of 10 K h^-1^. Block purple crystals obtained were washed with water and dried in air (yield: 45% based on Co).

### Refinement

H atoms on C atoms were placed at calculated positions and treated as riding atoms, with C—H = 0.93 Å and with *U*_iso_(H) = 1.2*U*_eq_(C). Water H atoms were tentatively located in difference Fourier maps and were refined with distance restraints of O—H = 0.82 (1) and H···H = 1.39 (1) Å and with *U*_iso_(H) = 1.5*U*_eq_(O). The highest peak is located 0.28 Å from H12A and the deepest hole is located 0.90 Å from Co1.

### Crystal data

**Table:**

[Co(CHO_2_)_2_(C_20_H_14_N_4_)(H_2_O)_2_]	*F*(000) = 1020
*M**_r_* = 495.35	*D*_x_ = 1.643 Mg m^−^^3^
Monoclinic, *C*2/*c*	Mo *K*α radiation, λ = 0.71073 Å
Hall symbol: -C 2yc	Cell parameters from 2895 reflections
*a* = 24.412 (5) Å	θ = 2.4–27.9°
*b* = 11.073 (2) Å	µ = 0.91 mm^−^^1^
*c* = 7.4117 (15) Å	*T* = 293 K
β = 91.28 (3)°	Block, purple
*V* = 2003.0 (7) Å^3^	0.30 × 0.26 × 0.21 mm
*Z* = 4	

### Data collection

**Table:**

Rigaku/MSC Mercury CCD diffractometer	2303 independent reflections
Radiation source: fine-focus sealed tube	1544 reflections with *I* > 2σ(*I*)
graphite	*R*_int_ = 0.063
ω scans	θ_max_ = 27.5°, θ_min_ = 3.1°
Absorption correction: multi-scan (*CrystalStructure*; Rigaku/MSC, 2002)	*h* = −31→31
*T*_min_ = 0.075, *T*_max_ = 0.126	*k* = −14→14
9417 measured reflections	*l* = −9→8

### Refinement

**Table:**

Refinement on *F*^2^	Primary atom site location: structure-invariant direct methods
Least-squares matrix: full	Secondary atom site location: difference Fourier map
*R*[*F*^2^ > 2σ(*F*^2^)] = 0.044	Hydrogen site location: inferred from neighbouring sites
*wR*(*F*^2^) = 0.112	H atoms treated by a mixture of independent and constrained refinement
*S* = 1.11	*w* = 1/[σ^2^(*F*_o_^2^) + (0.040*P*)^2^ + 3.*P*] where *P* = (*F*_o_^2^ + 2*F*_c_^2^)/3
2303 reflections	(Δ/σ)_max_ < 0.001
160 parameters	Δρ_max_ = 0.76 e Å^−^^3^
3 restraints	Δρ_min_ = −0.57 e Å^−^^3^

### Fractional atomic coordinates and isotropic or equivalent isotropic displacement parameters (Å^2^)

**Table:**

	*x*	*y*	*z*	*U*_iso_*/*U*_eq_	
Co1	0.2500	1.2500	0.0000	0.02543 (18)	
O1W	0.30131 (10)	1.13065 (19)	0.1536 (3)	0.0321 (5)	
H1W	0.2998 (17)	1.132 (3)	0.2643 (14)	0.048*	
H2W	0.3056 (16)	1.0629 (15)	0.110 (4)	0.048*	
O1	0.22769 (10)	1.35749 (18)	0.2125 (3)	0.0329 (5)	
O2	0.18853 (10)	1.3927 (2)	0.4744 (3)	0.0384 (6)	
N1	0.17914 (11)	1.1358 (2)	0.0338 (3)	0.0271 (6)	
N2	0.0000	0.9831 (3)	0.2500	0.0285 (8)	
N3	0.0000	0.3497 (4)	0.2500	0.0466 (11)	
C1	0.13056 (14)	1.1840 (3)	0.0711 (4)	0.0308 (7)	
H1	0.1269	1.2675	0.0632	0.037*	
C2	0.08571 (14)	1.1183 (3)	0.1202 (4)	0.0300 (7)	
H2	0.0528	1.1564	0.1458	0.036*	
C3	0.09050 (13)	0.9933 (3)	0.1310 (4)	0.0255 (7)	
C4	0.13993 (14)	0.9416 (3)	0.0889 (4)	0.0303 (7)	
H4	0.1444	0.8583	0.0929	0.036*	
C5	0.18262 (14)	1.0151 (3)	0.0409 (4)	0.0290 (7)	
H5	0.2157	0.9791	0.0117	0.035*	
C6	0.04323 (13)	0.9200 (3)	0.1906 (4)	0.0266 (7)	
C7	0.04429 (12)	0.7978 (2)	0.1879 (4)	0.0221 (6)	
H7	0.0744	0.7564	0.1447	0.027*	
C8	0.0000	0.7374 (4)	0.2500	0.0286 (9)	
C9	0.0000	0.6031 (4)	0.2500	0.0325 (10)	
C10	−0.04717 (16)	0.5383 (3)	0.2075 (5)	0.0386 (8)	
H10	−0.0800	0.5778	0.1805	0.046*	
C11	−0.04431 (18)	0.4138 (3)	0.2061 (5)	0.0466 (10)	
H11	−0.0758	0.3716	0.1719	0.056*	
C12	0.20695 (14)	1.3244 (3)	0.3573 (4)	0.0297 (7)	
H12A	0.2052	1.2418	0.3790	0.036*	

### Atomic displacement parameters (Å^2^)

**Table:**

	*U*^11^	*U*^22^	*U*^33^	*U*^12^	*U*^13^	*U*^23^
Co1	0.0309 (3)	0.0234 (3)	0.0222 (3)	−0.0028 (3)	0.0045 (2)	−0.0005 (2)
O1W	0.0425 (15)	0.0286 (11)	0.0252 (11)	−0.0011 (10)	0.0021 (10)	0.0004 (9)
O1	0.0438 (15)	0.0292 (11)	0.0259 (11)	−0.0069 (10)	0.0077 (10)	−0.0032 (9)
O2	0.0456 (16)	0.0435 (13)	0.0265 (11)	0.0001 (11)	0.0083 (10)	−0.0030 (11)
N1	0.0292 (15)	0.0256 (12)	0.0267 (13)	−0.0016 (11)	0.0015 (11)	−0.0010 (11)
N2	0.027 (2)	0.0286 (18)	0.0300 (18)	0.000	0.0020 (16)	0.000
N3	0.061 (3)	0.031 (2)	0.048 (3)	0.000	0.008 (2)	0.000
C1	0.032 (2)	0.0239 (15)	0.0368 (17)	0.0017 (13)	0.0008 (14)	0.0000 (13)
C2	0.0281 (19)	0.0259 (15)	0.0361 (17)	0.0022 (13)	−0.0003 (13)	−0.0048 (13)
C3	0.0302 (18)	0.0239 (14)	0.0222 (14)	0.0012 (12)	−0.0013 (12)	−0.0034 (12)
C4	0.035 (2)	0.0271 (15)	0.0286 (15)	0.0027 (14)	0.0045 (13)	−0.0003 (13)
C5	0.0306 (19)	0.0278 (15)	0.0289 (15)	0.0000 (13)	0.0050 (13)	−0.0024 (13)
C6	0.0289 (18)	0.0274 (15)	0.0234 (14)	−0.0002 (13)	0.0010 (12)	−0.0017 (13)
C7	0.0202 (16)	0.0197 (12)	0.0267 (14)	0.0008 (11)	0.0039 (12)	−0.0011 (12)
C8	0.027 (2)	0.029 (2)	0.030 (2)	0.000	0.0002 (17)	0.000
C9	0.037 (3)	0.029 (2)	0.031 (2)	0.000	0.003 (2)	0.000
C10	0.040 (2)	0.0339 (17)	0.0419 (19)	−0.0055 (15)	−0.0007 (16)	0.0043 (15)
C11	0.057 (3)	0.0375 (19)	0.046 (2)	−0.0155 (18)	0.0002 (18)	0.0001 (17)
C12	0.0324 (19)	0.0323 (16)	0.0245 (14)	−0.0036 (14)	0.0025 (13)	−0.0002 (14)

### Geometric parameters (Å, °)

**Table:**

Co1—O1	2.057 (2)	C2—C3	1.392 (4)
Co1—O1^i^	2.057 (2)	C2—H2	0.9300
Co1—O1W^i^	2.133 (2)	C3—C4	1.377 (4)
Co1—O1W	2.133 (2)	C3—C6	1.486 (4)
Co1—N1^i^	2.162 (3)	C4—C5	1.375 (4)
Co1—N1	2.162 (3)	C4—H4	0.9300
O1W—H1W	0.82 (1)	C5—H5	0.9300
O1W—H2W	0.82 (1)	C6—C7	1.353 (4)
O1—C12	1.251 (3)	C7—C8	1.361 (3)
O2—C12	1.243 (4)	C7—H7	0.9300
N1—C1	1.335 (4)	C8—C7^ii^	1.361 (3)
N1—C5	1.341 (4)	C8—C9	1.487 (6)
N2—C6	1.348 (4)	C9—C10	1.387 (4)
N2—C6^ii^	1.348 (4)	C9—C10^ii^	1.387 (4)
N3—C11^ii^	1.328 (5)	C10—C11	1.381 (5)
N3—C11	1.328 (5)	C10—H10	0.9300
C1—C2	1.371 (4)	C11—H11	0.9300
C1—H1	0.9300	C12—H12A	0.9300
			
O1—Co1—O1^i^	180.0	C4—C3—C2	118.2 (3)
O1—Co1—O1W^i^	83.56 (9)	C4—C3—C6	122.1 (3)
O1^i^—Co1—O1W^i^	96.44 (9)	C2—C3—C6	119.7 (3)
O1—Co1—O1W	96.44 (9)	C5—C4—C3	119.1 (3)
O1^i^—Co1—O1W	83.56 (9)	C5—C4—H4	120.5
O1W^i^—Co1—O1W	180.0	C3—C4—H4	120.5
O1—Co1—N1^i^	88.67 (9)	N1—C5—C4	123.5 (3)
O1^i^—Co1—N1^i^	91.33 (9)	N1—C5—H5	118.2
O1W^i^—Co1—N1^i^	92.16 (9)	C4—C5—H5	118.2
O1W—Co1—N1^i^	87.84 (9)	N2—C6—C7	122.6 (3)
O1—Co1—N1	91.33 (9)	N2—C6—C3	115.6 (3)
O1^i^—Co1—N1	88.67 (9)	C7—C6—C3	121.7 (3)
O1W^i^—Co1—N1	87.84 (9)	C6—C7—C8	118.1 (3)
O1W—Co1—N1	92.16 (9)	C6—C7—H7	121.0
N1^i^—Co1—N1	180.0	C8—C7—H7	121.0
Co1—O1W—H1W	119 (2)	C7—C8—C7^ii^	121.1 (4)
Co1—O1W—H2W	116 (2)	C7—C8—C9	119.5 (2)
H1W—O1W—H2W	114.8 (18)	C7^ii^—C8—C9	119.5 (2)
C12—O1—Co1	127.3 (2)	C10—C9—C10^ii^	117.7 (4)
C1—N1—C5	116.5 (3)	C10—C9—C8	121.2 (2)
C1—N1—Co1	120.5 (2)	C10^ii^—C9—C8	121.2 (2)
C5—N1—Co1	122.5 (2)	C11—C10—C9	118.4 (4)
C6—N2—C6^ii^	117.5 (4)	C11—C10—H10	120.8
C11^ii^—N3—C11	115.4 (4)	C9—C10—H10	120.8
N1—C1—C2	124.1 (3)	N3—C11—C10	125.0 (4)
N1—C1—H1	117.9	N3—C11—H11	117.5
C2—C1—H1	117.9	C10—C11—H11	117.5
C1—C2—C3	118.5 (3)	O2—C12—O1	125.5 (3)
C1—C2—H2	120.8	O2—C12—H12A	117.2
C3—C2—H2	120.8	O1—C12—H12A	117.2

Symmetry codes: (i) −*x*+1/2, −*y*+5/2, −*z*; (ii) −*x*, *y*, −*z*+1/2.

### Hydrogen-bond geometry (Å, °)

**Table:**

*D*—H···*A*	*D*—H	H···*A*	*D*···*A*	*D*—H···*A*
O1W—H1W···O2^iii^	0.82 (1)	1.97 (1)	2.775 (3)	166 (4)
O1W—H2W···O2^iv^	0.82 (1)	1.99 (1)	2.814 (3)	174 (3)
C2—H2···N3^v^	0.93	2.62	3.458 (3)	150
C5—H5···O2^iv^	0.93	2.53	3.429 (4)	164

Symmetry codes: (iii) −*x*+1/2, −*y*+5/2, −*z*+1; (iv) −*x*+1/2, *y*−1/2, −*z*+1/2; (v) *x*, *y*+1, *z*.
